# Gene model for the ortholog of *mts* in *Drosophila mojavensis*

**DOI:** 10.17912/micropub.biology.000888

**Published:** 2025-02-17

**Authors:** Megan E. Lawson, Clairine I. S. Larsen, Madeline McAbee, Scott Tanner, Jeffrey S. Thompson, Chinmay P. Rele, Laura K Reed

**Affiliations:** 1 University of Alabama, Tuscaloosa, AL USA; 2 Denison University, Granville, OH USA; 3 University of South Carolina Upstate, Spartanburg, SC USA

## Abstract

Gene model for the ortholog of *microtubule star* ( *mts* ) in the D. mojavensis May 2011 (Agencourt dmoj_caf1/DmojCAF1) Genome Assembly (GenBank Accession: GCA_000005175.1 ) of *Drosophila mojavensis* . This ortholog was characterized as part of a developing dataset to study the evolution of the Insulin/insulin-like growth factor signaling pathway (IIS) across the genus *Drosophila* using the Genomics Education Partnership gene annotation protocol for Course-based Undergraduate Research Experiences.

**Figure 1. mts gene model comparison between Drosophila mojavensis and Drosophila melanogaster orthologs:**
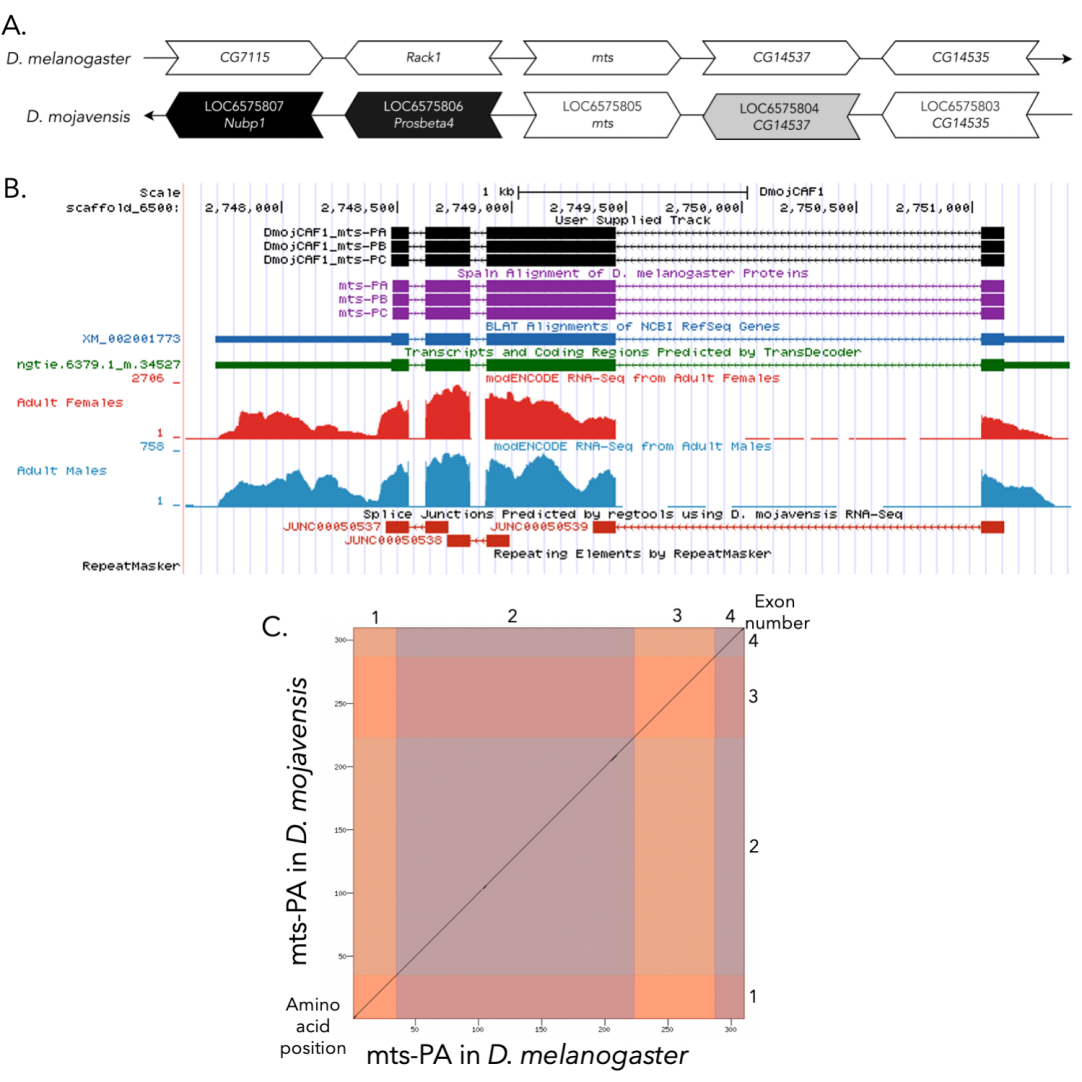
(A) Synteny of genomic neighborhood of *mts* in *D. melanogaster* and *D. mojavensis* . Gene arrows pointing in the same direction as reference gene in both *D. mojavensis* and *D. melanogaster* are on the same strand as the reference gene; gene arrows pointing in the opposite direction are on the opposite strand. The thin underlying arrows pointing to the right indicate that *mts* is on the + strand; arrows pointing to the left indicate that *mts* is on the – strands. Arrows in *D. mojavensis* indicate the locus ID of the genes, white arrows indicate orthology to the corresponding gene in *D. melanogaster* , gray arrows indicate an orthologous gene pointing in the opposite direction as *D. melanogaster* relative to *mts* , and black arrows indicate a non-orthologous gene. The gene names given in the *D. mojavensis* gene arrows indicate the orthologous gene in *D. melanogaster* , while the locus identifiers are specific to *D. mojavensis* **.** (B) Gene Model in UCSC Track Hub (Raney et al. 2014): the gene model in *D. mojavensis* (black), Spaln of *D. melanogaster* Proteins (purple, alignment of RefSeq proteins from *D. melanogaster* ), BLAT alignments of NCBI RefSeq Genes (blue, alignment of RefSeq genes for *D. mojavensis* ), RNA-Seq from adult females (red) and adult males (blue) (alignment of Illumina RNA-Seq reads from *D. mojavensis* ), and Transcripts (green) including coding regions predicted by TransDecoder and Splice Junctions Predicted by regtools using *D. mojavensis* RNA-Seq (Chen *et al.* , 2014; SRP006203 ). Splice junctions shown have a minimum read-depth of 1362 with >1000 supporting reads shown in red. The custom gene model (User Supplied Track) is indicated in black with CDSs depicted with boxes and introns with narrow lines (arrows indicate direction of transcription). (C) Dot Plot of mts_PA in *D. melanogaster* ( *x* -axis) vs. the orthologous peptide in *D. mojavensis* ( *y* -axis). Amino acid number is indicated along the left and bottom; CDS number is indicated along the top and right, and CDSs are also highlighted with alternating colors.

## Description

**Table:** 

* This article reports a predicted gene model generated by undergraduate work using a structured gene model annotation protocol defined by the Genomics Education Partnership (GEP; thegep.org) for Course-based Undergraduate Research Experience (CURE). The following information may be repeated in other articles submitted by participants using the same GEP CURE protocol for annotating Drosophila species orthologs of Drosophila melanogaster genes in the insulin signaling pathway. * "In this GEP CURE protocol students use web-based tools to manually annotate genes in non-model *Drosophila* species based on orthology to genes in the well-annotated model organism fruitfly *Drosophila melanogaster* . The GEP uses web-based tools to allow undergraduates to participate in course-based research by generating manual annotations of genes in non-model species (Rele et al., 2023) . Computational-based gene predictions in any organism are often improved by careful manual annotation and curation, allowing for more accurate analyses of gene and genome evolution (Mudge and Harrow 2016; Tello-Ruiz et al., 2019) . These models of orthologous genes across species, such as the one presented here, then provide a reliable basis for further evolutionary genomic analyses when made available to the scientific community.” (Myers et al., 2024) . “The particular gene ortholog described here was characterized as part of a developing dataset to study the evolution of the Insulin/insulin-like growth factor signaling pathway (IIS) across the genus *Drosophila* . The Insulin/insulin-like growth factor signaling pathway (IIS) is a highly conserved signaling pathway in animals and is central to mediating organismal responses to nutrients (Hietakangas and Cohen 2009; Grewal 2009) .” (Myers et al., 2024) . “ *D.* *mojavensis* (NCBI:txid7230) is part of the *mulleri complex* in the *repleta* species group within the subgenus *Drosophila* of the genus *Drosophila* (Wasserman 1992; Durando et al., 2000) *.* It was first described by Patterson (Patterson and Crow 1940) . *D. mojavensis* specializes on rotting cactus as its host and is found in the Mojave and Sonoran Deserts of the southwestern United States and northwestern Mexico including the Baja Peninsula, as well as on the channel-islands off the coast of California (https://www.taxodros.uzh.ch, accessed 1 Feb 2023).” (Congleton et al., 2022) . “The *mts* ( *microtubule star* , protein phosphatase 2A, PP2A, PP2A-C, PP2Ac, PP2A catalytic subunit) gene encodes the catalytic subunit of protein phosphatase 2A, which can inhibit IIS by dephosphorylating the insulin receptor and protein kinase B (AKT) (Vereshchagina et al., 2008; Gil-Ranedo et al., 2019; Yuan et al., 2020; Chew et al., 2022). mts deficiency in *Drosophila* causes disorganization of microtubules into a star-like pattern, ultimately leading to a fatal condensing of chromatin, halting mitosis early in development (Snaith et al., 1996) . mts is involved in other processes in *Drosophila* , including regulating hedgehog and Wnt/Wingless signaling (Mayer-Jaekel et al., 1992; Casso et al., 2008; Jia et al., 2009; Zhang et al., 2009; Su et al., 2011).” (Lawson et al., 2025) .

The model presented here is the ortholog of *mts* in the May 2011 (Agencourt dmoj_caf1/DmojCAF1) assembly of *D. mojavensis* ( GCA_000005175.1 ) and corresponds to the Gnomon Peptide ID ( XM_002001773 ) predicted model in *D. mojavensis* ( LOC6575805 ) *.* This gene model is based on RNA-Seq data from *D. mojavensis* (Chen et al *.* , 2014; SRP006203 *) and the mts* in *D. melanogaster* from FB2023_03 ( GCA_000001215.4 ; Larkin et al., 2021; Gramates et al., 2022; Jenkins et al., 2022).

***Synteny***

*mts* occurs on chromosome 2L in *D. melanogaster* and is flanked upstream by *Rack1* and *CG7115* and downstream by *CG14537* and *CG14535 .* We determined that the putative ortholog of *mts* is found on scaffold 6500 ( CH933807.1 ) in *D. mojavensis* (CAF1 assembly GCA_000005175.1 ) with LOC6575805 ( XP_002001809.1 ) (via *tblastn* search with an e-value of 0.0 and percent identity of 99.68%). It is flanked upstream by LOC6575807 ( XP_002001811.1 ) and LOC6575806 ( XP_002001810.1 ), which correspond to *Nubp1*  and *Prosbeta4* in *D. melanogaster* with e-values of ­­­1e-78 and 1e-123 respectively and percent identities of 86.13% and 80.40% respectively, as determined by *blastp* . It is flanked downstream by LOC6575804 ( XP_002001808.1 ) and LOC6575803 ( XP_032584898.1 ), which correspond to *CG14537* and *CG14535* in *D. melanogaster* with e-values of ­­­8e-61 and 0.0 respectively and percent identities of 50.47% and 75.04% respectively as determined by *blastp* ( Figure 1A, A ltschul et al., 1990). We believe this is the correct ortholog assignment for *mts* in *D. mojavenis* because the *tblastn* hit for *mts* is very high quality, as it has a 99.68% identity and an e-value of 0.0. Additionally, the order of the downstream genes is conserved, although it is important to note that *CG14535* is on the same strand as *mts* in *D. melanogaster* but is on the opposite strand relative to *mts* in *D. mojavensis ,* so local synteny is not completely conserved in the downstream region. This inversion is also present in the sister species, *D. arizonae .* The upstream region is also different between the two species.

***Protein Model***

*mts* in *D. mojavensis* has one protein coding isoform (mts-PA, mts-PB, and mts-PC) ( Figure 1B ), encoded by mRNAs mts-RA, mts-RB, and mts-RC, which differ in their UTRs, that contain four CDSs. This is the same relative to the ortholog in *D. melanogaster* , which also has one protein coding isoform, encoded by four CDSs. The sequence of *mts* in *D. mojavensis* has 99.68% identity with the *mts* in *D. melanogaster* as determined by *blastp* ( Figure 1C ). The coordinates of the curated gene models can be found in NCBI at GenBank/BankIt using the accessions BK063009 , BK063010 , and BK063011 . These data are also available in Extended Data files below, which are archived in CaltechData.

## Methods

Detailed methods including algorithms, database versions, and citations for the complete annotation process can be found in Rele et al. (2023). Briefly, students use the GEP instance of the UCSC Genome Browser v.435 (https://gander.wustl.edu ; Kent WJ et al., 2002; Navarro Gonzalez et al., 2021) to examine the genomic neighborhood of their reference IIS gene in the *D. melanogaster* genome assembly (Aug. 2014; BDGP Release 6 + ISO1 MT/dm6). Students then retrieve the protein sequence for the *D. melanogaster* reference gene for a given isoform and run it using tblastn against their target *Drosophila* species genome assembly ( *D. mojavensis* ( GCA_000005175.1 )) on the NCBI BLAST server (https://blast.ncbi.nlm.nih.gov/Blast.cgi, Altschul et al., 1990) to identify potential orthologs. To validate the potential ortholog, students compare the local genomic neighborhood of their potential ortholog with the genomic neighborhood of their reference gene in *D. melanogaster* . This local synteny analysis includes at minimum the two upstream and downstream genes relative to their putative ortholog. They also explore other sets of genomic evidence using multiple alignment tracks in the Genome Browser, including BLAT alignments of RefSeq Genes, Spaln alignment of *D. melanogaster* proteins, multiple gene prediction tracks (e.g., GeMoMa, Geneid, Augustus), and modENCODE RNA-Seq from the target species. Detailed explanation of how these lines of genomic evidenced are leveraged by students in gene model development are described in Rele et al. (2023). Genomic structure information (e.g., CDSs, CDS number and boundaries, number of isoforms) for the *D. melanogaster* reference gene is retrieved through the Gene Record Finder (https://gander.wustl.edu/~wilson/dmelgenerecord/index.html; Rele et al., 2023). Approximate splice sites within the target gene are determined using tblastn using the CDSs from the *D. melanogaster* reference gene. Coordinates of CDSs are then refined by examining aligned modENCODE RNA-Seq data, and by applying paradigms of molecular biology such as identifying canonical splice site sequences and ensuring the maintenance of an open reading frame across hypothesized splice sites. Students then confirm the biological validity of their target gene model using the Gene Model Checker (https://gander.wustl.edu/~wilson/dmelgenerecord/index.html; Rele et al., 2023), which compares the structure and translated sequence from their hypothesized target gene model against the *D. melanogaster* reference gene model. At least two independent models for this gene were generated by students under mentorship of their faculty course instructors. These models were then reconciled by a third independent researcher mentored by the project leaders to produce the final model presented here. Note: comparison of 5' and 3' UTR sequence information is not included in this GEP CURE protocol.

## Data Availability

Description: GFF, FASTA, and PEP of the model. Resource Type: Model. DOI: https://doi.org/10.22002/ebd92-xae03
